# Characterization of a novel *MYO3A* missense mutation associated with a dominant form of late onset hearing loss

**DOI:** 10.1038/s41598-018-26818-2

**Published:** 2018-06-07

**Authors:** Vitor G. L. Dantas, Manmeet H. Raval, Angela Ballesteros, Runjia Cui, Laura K. Gunther, Guilherme L. Yamamoto, Leandro Ucela Alves, André Silva Bueno, Karina Lezirovitz, Sulene Pirana, Beatriz C. A. Mendes, Christopher M. Yengo, Bechara Kachar, Regina C. Mingroni-Netto

**Affiliations:** 1 Centro de Pesquisas sobre o Genoma Humano e Células-Tronco, Departamento de Genética e Biologia Evolutiva, Instituto de Biociências, Universidade de São Paulo, São Paulo, Brazil; 2 Department of Cellular and Molecular Physiology, Pennsylvania State University, College of Medicine, Hershey, PA 17033 USA; 3 Laboratory of Cell Structure and Dynamics, National Institute on Deafness and Other Communication Disorders, National Institutes of Health, Bethesda, MD 20892 USA; 4 Laboratório de Otorrinolaringologia/LIM32 – Hospital das Clínicas, Faculdade de Medicina, Universidade de São Paulo, São Paulo, Brazil; 5 UNIFAL - Universidade Federal de Alfenas, Alfenas, Brazil; 6 Universidade São Francisco, Braganca Paulista, Brazil; 7 Divisão de Educação e Reabilitação dos Distúrbios da Comunicação da Pontifícia Universidade Católica de São Paulo, São Paulo, Brazil

## Abstract

Whole-exome sequencing of samples from affected members of two unrelated families with late-onset non-syndromic hearing loss revealed a novel mutation (c.2090 T > G; NM_017433) in *MYO3A*. The mutation was confirmed in 36 affected individuals, showing autosomal dominant inheritance. The mutation alters a single residue (L697W or p.Leu697Trp) in the motor domain of the stereocilia protein MYO3A, leading to a reduction in ATPase activity, motility, and an increase in actin affinity. MYO3A-L697W showed reduced filopodial actin protrusion initiation in COS7 cells, and a predominant tipward accumulation at filopodia and stereocilia when coexpressed with wild-type MYO3A and espin-1, an actin-regulatory MYO3A cargo. The combined higher actin affinity and duty ratio of the mutant myosin cause increased retention time at stereocilia tips, resulting in the displacement of the wild-type MYO3A protein, which may impact cargo transport, stereocilia length, and mechanotransduction. The dominant negative effect of the altered myosin function explains the dominant inheritance of deafness.

## Introduction

Hearing loss is the most frequent sensory disability, affecting almost 360 million people of different ages (World Health Organization, WHO, 2012). Multiple causative factors including genetic, environmental, or a combination of both give rise to enormous heterogeneity associated with hearing loss. According to Keats and Berlin (1999), roughly 70% of the hearing loss cases lack additional clinical signs and are categorized as non-syndromic hearing loss^1^. Autosomal recessive (DFNB) inheritance is the most common (80% cases) pattern of hearing loss inheritance in genetic cases (www.iro.es/cx26deaf.html), whereas only 10–20% of the cases correspond to an autosomal dominant (DFNA) mechanism^2^. Previous studies have mapped approximately 170 loci for non-syndromic hearing loss out of which, only half of the genes have been identified (http://hereditaryhearingloss.org). It is important to note that, in addition to extensive locus heterogeneity and allelic diversity within the same locus, some genetic mutations lead to a recessive hearing loss while other mutations in the same gene lead to a dominant hearing loss. Most of these mutations lead to an early onset hearing loss phenotype, whereas other mutations result in dominant inheritance and late onset phenotypes. There is a lack of knowledge about the mechanisms of dominant inheritance and/or late onset hearing loss of genetic origin, thus any clinical data related to such phenotypes are vital to understand the molecular basis of hearing loss progression. Moreover, insights into the mechanism can be crucial to develop therapies for late-onset hearing loss cases which may be more amenable to corrective treatments.

Vertebrate class III myosins (MYO3) consists of two isoforms encoded by separate genes, myosin IIIA (*MYO3A*) and myosin IIIB (*MYO3B*). The *MYO3A* gene was mapped to locus DFNB30 in a three-generation Jewish family with individuals affected by autosomal recessive nonsyndromic hearing loss^3^. NINAC (neither inactivation nor activation potential C) was the first member of MYO3 to be discovered in a *Drosophila* mutant screen^4,5^. NINAC null mutant flies demonstrated a defective phototransduction cascade^6^. Members of the unconventional myosin superfamily, class III myosins uniquely have a kinase domain at their N-terminus, followed by a conserved motor domain, two IQ motifs and a class specific C-terminal tail region^7,8^. Vertebrate MYO3 proteins are actin-dependent motor proteins that move towards the plus end of actin filaments^9–11^. MYO3 dependent transport of espin-1 (ESPN-1) and espin like (ESPNL) from the base to the tips of hair cell stereocilia is thought to be crucial for stereocilia length regulation and maintenance of the staircase-shaped stereocilia bundle organization^12,13^. Class III myosin isoforms contain a conserved ESPN isoform binding site called tail homology I motif (THD1) in their tail domain^12–14^. However, MYO3A has an unique extended tail domain consisting of an actin binding site called tail homology II motif (THD2)^10^, as well as a binding site for membrane occupation nexus motif protein (Morn4)^15^. It was recently reported that MYO3A may also be involved in the transport of Protocadherin-15, an essential component of the mechano-electrical transduction (MET) complex in stereocilia^16^.

A mouse model harboring human *MYO3A* mutations (DFNB30) demonstrated significant hearing loss at 2.5 months of age, beginning first at high frequencies and eventually producing hearing loss at all frequencies^17^. Recently, two studies reported novel *MYO3A* mutations associated with non-syndromic hearing loss. The first report revealed an autosomal recessive mutation in a highly conserved residue of the MYO3A motor domain (p.Ser614Phe) from a consanguineous Kazakh family with a congenital hearing-loss phenotype^18^. The second report characterized an autosomal dominant mutation (p.Gly488Glu) in the MYO3A motor domain associated with progressive hearing loss in a two-generation African American family^16^. However, the mechanism of the impact of *MYO3A* mutants p.Ser614Phe and p.Gly488Glu on stereocilia structure and function is not well established.

In the current study, we carried out massive parallel whole-exome sequencing of affected individuals from two large unrelated Brazilian families presenting autosomal dominant hearing loss and identified a novel mutation in *MYO3A* on 26 affected members from family 1 and 10 affected individuals from family 2. Thus, this report is the second to implicate *MYO3A* in autosomal dominant hearing loss, suggesting an essential role for this protein in hearing. The novelty of the mutation and the lack of understanding about *MYO3A* associated autosomal dominant hearing loss, motivated us to investigate the molecular basis of the unusual dominant and late-onset phenotype. We hypothesized that the mutant MYO3A would impair the function of the wild-type MYO3A in the stereocilia of inner ear hair cells. We performed a complete functional characterization of the mutant protein using biochemical/biophysical assays of the purified MYO3A motor. Additionally, we performed cell biological analysis of the localization of the mutant MYO3A in actin-based protrusions known as filopodia and in stereocilia. Our results allowed us to propose a model for how the mutation leads to a dominant negative impact on the function of MYO3A in inner ear hair cell stereocilia.

## Results

### Clinical findings

**Figure 1:**
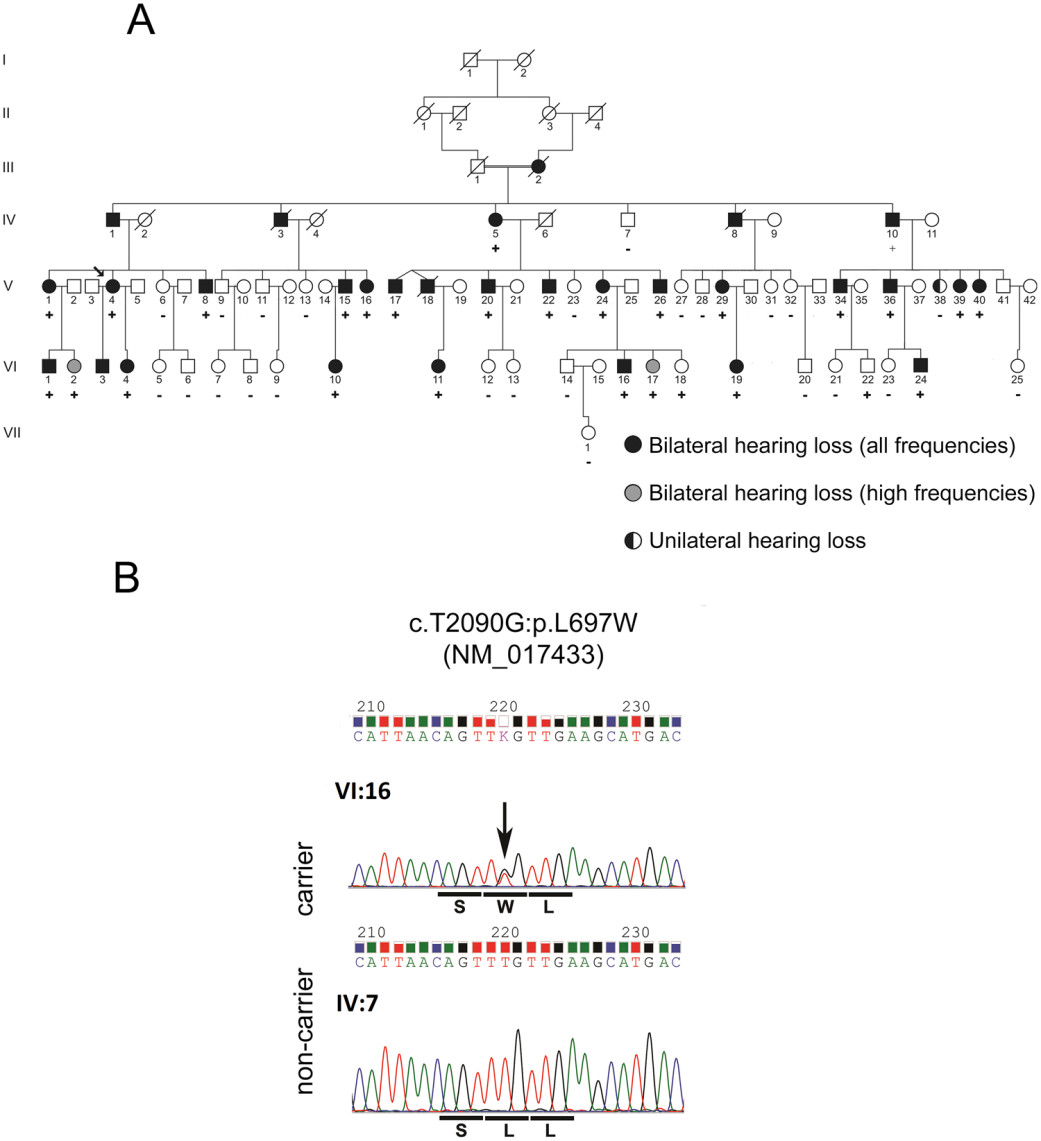
Novel *MYO3A* dominant mutation leads to hearing loss. (**A**) Simplified pedigree of family 1 showing individuals whose samples were tested for the presence of the c.2090 T > G mutation in *MYO3A*. Individuals indicated with a plus sign (+) are heterozygotes for the c.2090 T > G mutation and individuals indicated with a minus sign (−) do not carry the mutation. (**B**) Chromatogram showing the c.2090 T > G variant in heterozygosis in individual VI:16 and the wildtype sequence in individual IV-7 from family 1.

**Figure 2:**
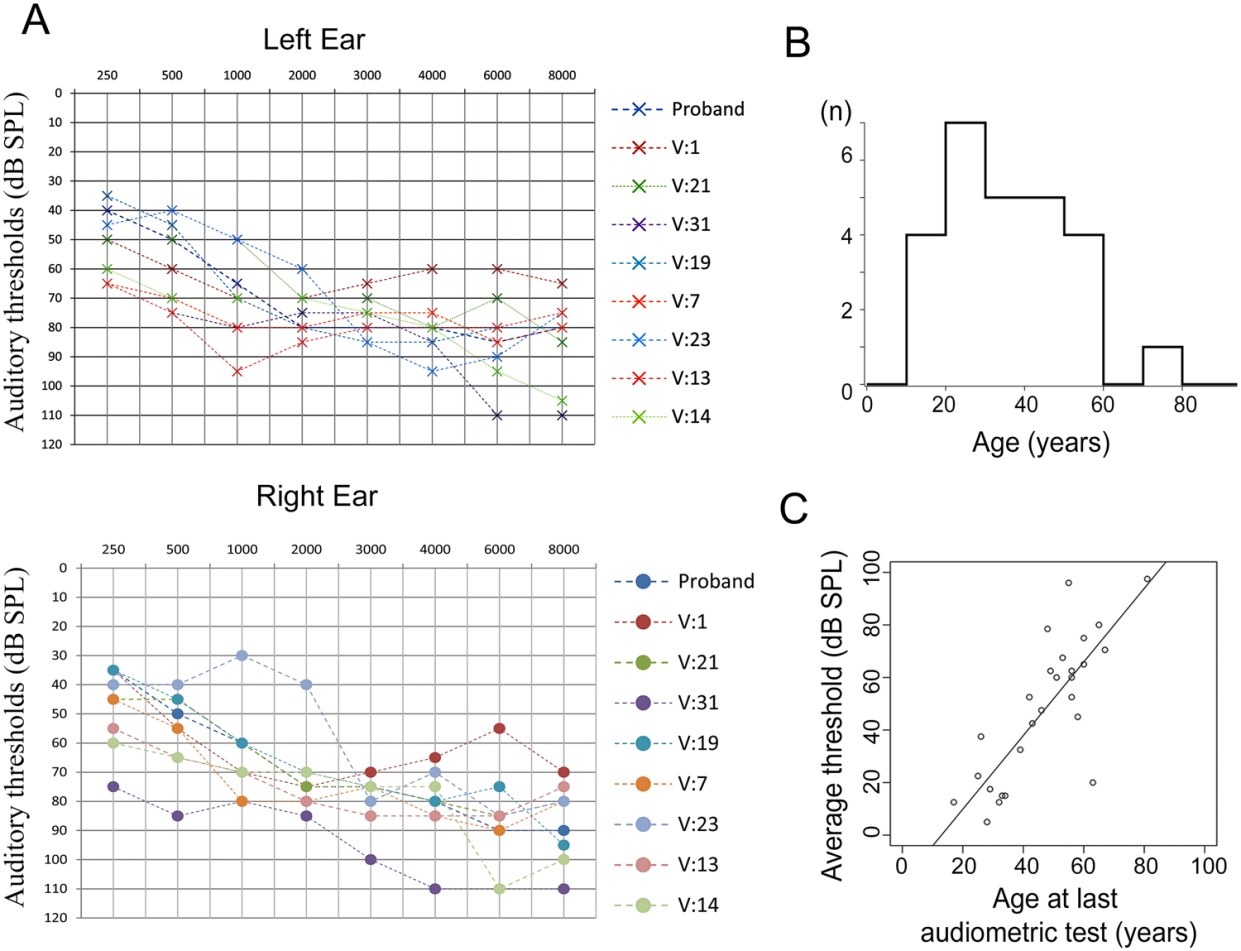
Tonal Audiometry thresholds of affected family members. (**A**) Tonal audiometry thresholds of nine affected individuals from family 1. (**B**) Age distribution of onset of hearing loss in family 1. (**C**) Distribution of average thresholds from both ears of the affected individuals of family 1 according to their age at examination showing that the fit to a linear regression model is excellent (r^2^ = 0.6173; t = 6.68; d.f. = 26; p < 10-5).

We identified and performed audiological evaluations in individuals from two unrelated families from the southeastern region of Brazil presenting hearing loss (Figs. 1A and S1A). Hearing loss in both families can be described as nonsyndromic sensorineural bilateral and progressive, affecting all frequencies ranging from mild to severe (Figs. 2A and S1B). In family 1, the age of onset varied from 12 to 70 years, with an average age of onset of 32 years, as reported by the patients (Fig. 2B). Family 2 presented a congenital hearing loss case, and the age of onset ranged from 0 to 36 years. If the congenital case is excluded, the average age of onset in family 2 is 30 years (data not shown). In addition, we examined the average audiometric thresholds and age of 28 carriers of the novel *MYO3A* variant from family 1 (Fig. 2C). Our data revealed a clear correlation between age and audiometric thresholds, which is expected in progressive hearing loss. Tinnitus was reported by 63% of affected individuals in family 1 and by 80% of affected individuals in family 2, being a frequent common complaint in both families.

### Whole-exome sequencing and segregation of candidate mutations

We collected 52 samples from affected and unaffected individuals from family 1. Whole exome sequencing was performed in four samples of this family with paired-end reads of 99 base pairs, to an average coverage of 120× per individual. All exonic or splicing variants present in the four affected individuals, that were not detected in 62 Brazilian whole-exome sequencing control samples, were screened for quality and checked against the 1000 Genomes Project public variant database (http://www.1000genomes.org/), the 6500 exomes database from Washington University (http://evs.gs.washington.edu/EVS/) and the Brazilian ABraOM database (http://abraom.ib.usp.br). Sixteen variants with a frequency of less than 1% in control populations were identified, however, because of the dominant pattern in the pedigree, only the four variants that have never been reported in controls (1000 Genomes, 6500 ESP exomes, and ABraOM) were selected for primary analysis (Table S1). Two of the four variants were selected first for segregation studies because the candidate genes could be functionally related to hearing loss. The variant c.1543 C > T: p.Gln515*, 16:48.333.581 in gene *LONP2* (NM_031490) is a nonsense mutation. The variant c.2090 T > G: p.Leu697Trp, [hg19]chr10:26.414.513 in the gene *MYO3A* (NM_017433.4) is a nonsynonymous substitution mutation, in a gene that has already been related to autosomal recessive hearing loss. Polyphen2 predictions indicated a probability of being damaging of 98.9% (score of 0.989) for p.Leu697Trp in MYO3A. SIFT and Mutation Taster also provided a damaging prediction (scores of 1 and 0.0001, respectively).

All 52 samples collected from family 1 were screened by Sanger sequencing for the presence of the variants c.2090 T > G(*MYO3A*) and c.1543 C > T(*LONP2*). The variant in the *LONP2* gene does not co-segregate with the phenotype, but the variant in the *MYO3A* gene was shown to correlate with the phenotype and it was present in 26 of 27 affected individuals. It was absent in one exceptional affected individual presenting unilateral hearing loss (V-38, Fig. 1A), who reported the onset of hearing loss on the right ear after infection at the age of six. In addition, the *MYO3A* variant was found in two unaffected individuals (VI-18; VI-22, Fig. 1A) who are below the average age of onset (28 and 29). Two-point Lod scores were calculated, one point being the phenotype of hearing loss and the second being the presence of the variant c.2090 T > G. The Lod score obtained with Merlin software was 9.50.

A similar strategy of filtering variants was applied to the four samples of family 2. A total of 75 exonic or splicing variants were present in the four affected individuals, with a frequency <1% in 1000 Genomes Project public variant database, the 6500 exomes database from Washington University, and the Brazilian ABraOM database. Once again, only 4 variants were not present in the control population and among those there was the same variant in *MYO3A* identified in family 1 (Table S2). Since the segregation of hearing loss with variant c.2090 T > G (*MY03A*) was confirmed in family 1, its transmission in family 2 was promptly investigated. It was found to be present in 10 affected patients and was detected in only one of the 10 unaffected individuals (IV-11, Fig. S1), who is 30 years old. This individual presents normal thresholds, but complains of tinnitus. Another exceptional affected individual without the mutation was found in family 2 (IV-2, Fig. S1) and noise induced hearing loss was suspected as the cause of hearing loss. A Lod score of 3.31 was obtained in family 2.

Inspection of the seven-generation pedigree of family 1 did not show any link to family members of family 2. To examine the possibility of kinship between the individuals of the two pedigrees, IBD analyses was performed in pairs, including 5 individuals from family 1 and 5 individuals from family 2, genotyped with the SNP array - Axiom Human Origins. In 16 pairs of individuals, there was no evidence of a close relationship. However, in 9 pairs, the kinship coefficient was equivalent to second cousins (Table S2). The examination of shared haplotypes revealed all 10 individuals share a 560.878pb haplotype that includes 131 SNPs. These data indicate the mutation probably has a common origin in the two families, who are remotely related.

One sample from an affected individual from family 1 (V-24) and one sample from an affected individual of family 2 (III-22) were analyzed by array-CGH and results allowed for exclusion of pathogenic copy number variants.

### Impact of the L697W mutation on MYO3A ATPase and *in vitro* motility

**Figure 3:**
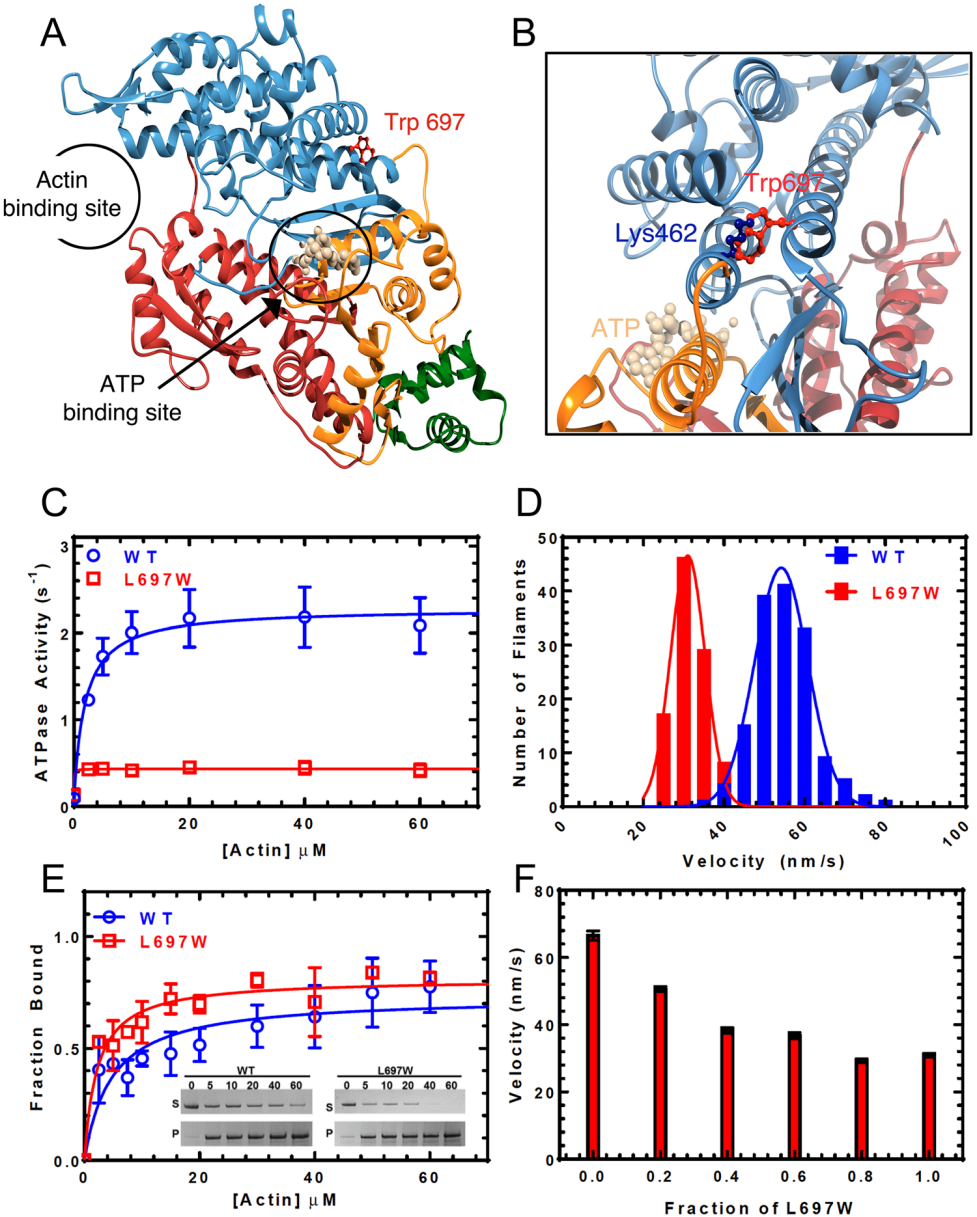
L697W mutation localization and effect on MYO3A motor properties. (**A**) Homology model of MYO3A motor domain in ribbon representation with the N-terminal domain in orange, U50 in blue, L50 in red and the converter domain in green. The ATP-binding site and actin-binding pocket are indicated. The ATP molecule bound to the nucleotide-binding site is shown in ball and stick representation and colored in tan. Trp697 is labeled, colored in red and its side chain is shown in ball and stick representation. (**B**) Close up view of Trp697 and Lys462 side chains position and clash. (**C**) The steady state actin-activated ATPase activity was plotted as a function of actin concentration and the data were fit to a hyperbolic function to determine maximum ATPase activity (k_cat_) and the actin concentration at which ATPase is one-half maximal (K_ATPase_). (**D**) The *in vitro* motility of the WT and L697W MYO3A was compared (n = 150 filaments). The actin sliding velocities for each construct were fit to a Gaussian distribution and the average velocity was determined. (**E**) Actin co-sedimentation assays were performed to examine the steady state actin affinity (K_Actin_). The fraction bound is plot a function of actin concentration and fit to a hyperbolic function to determine K_Actin_. (Inset shows the coomassie stained SDS-PAGE gels used to resolve the fraction bound at each actin concentration). (**F**) *In vitro* motility examined in a mixture of L697W and WT MYO3A. The average velocity of 25 filaments per condition is plotted in each condition (fraction of mutant to total MYO3A present). Table 1 displays the summary of ATPase and *in vitro* motility values.

The novel mutation identified in this study causes a tryptophan to replace leucine residue 697, a residue that is conserved between MYO3 isoforms but not conserved in other myosins (Fig. S2). The L697W mutation maps in the motor domain of MYO3A (amino acids 340-988 of NM_017433.4). To localize the L697W mutation in the MYO3A motor domain structure, we generated a homology model using SWISS-MODEL^19^ and the human MYO10 structure (PDB ID: 5i0h) as a template (Fig. 3A, B). Leucine 697 is located in the upper 50KDa domain (U50) at the end of a long alpha helix within this domain, distant from the ATP binding pocket and the actin-binding region.

The impact of the L697W mutation on MYO3A biochemical and biophysical properties were examined using purified human MYO3A. The kinase domain of MYO3A is known to autophosphorylate the MYO3A motor and reduce motor activity^20^, which may have an impact on its cellular localization and function. To examine the effect of the L697W mutation on MYO3A without any influence of autoregulation, we used MYO3A constructs lacking the kinase domain (MYO3AΔK) for our biochemical and cell-biological experiments. We used C-terminal GFP tagged wild-type and L697W MYO3A constructs lacking the kinase domain and containing the motor and 2IQ domains (MYO3AΔK 2IQ c-GFP) for ATPase and *in vitro* motility experiments. The C-terminal GFP tag was used to attach the protein to the surface of coverslips coated with anti-GFP antibody to perform the *in vitro* motility assay. Our results show that the construct containing the mutation (MYO3AΔK 2IQ L697W) has a ~7 fold lower maximal ATPase rate and ~2 fold higher actin affinity (Table 1 and Fig. 3C, E) compared to the corresponding WT construct. The actin dependence of the ATPase activity (K_ATPase_) of the L697W mutant was reduced at least 2-fold indicating that the mutant reaches maximal ATPase activity at extremely low actin concentrations. We also observed that the mutant protein resulted in a ~2 fold decrease in the actin sliding velocity in the *in vitro* motility assay compared to the WT protein (Table 1 and Fig. 3D). The presence of the mutant MYO3A also reduced the velocity of WT MYO3A in a concentration-dependent manner as assessed using mixture motility assays (Fig. 3F). Overall, our results demonstrate that the mutation alters the enzymatic and motile properties of MYO3A.

**Table 1:** Summary of actin activated ATPase and *in vitro* motility results.

Construct	V_0_ (s^−1^)	k_cat_ (s^−1^)	K_ATPase_ (µM)	K_Actin_ (µM)	Velocity (nm/s)
MYO3A ΔK 2IQ WT	0.09 ± 0.01	2.19 ± 0.09	1.93 ± 0.48	4.69 ± 1.60	54.8 ± 0.6
MYO3A ΔK 2IQ L697W	0.13 ± 0.01	0.31 ± 0.01	≤0.77	2.21 ± 0.53	31.3 ± 0.4

ATPase data (represented as mean ± SEM) and *in vitro* motility data (represented as mean ± SEM; n = 150 filaments) were reported from at least 3 protein preparations.

### Characterization of the impact of the L697W mutation on MYO3A filopodia initiation and elongation activity in COS7 cells

**Figure 4:**
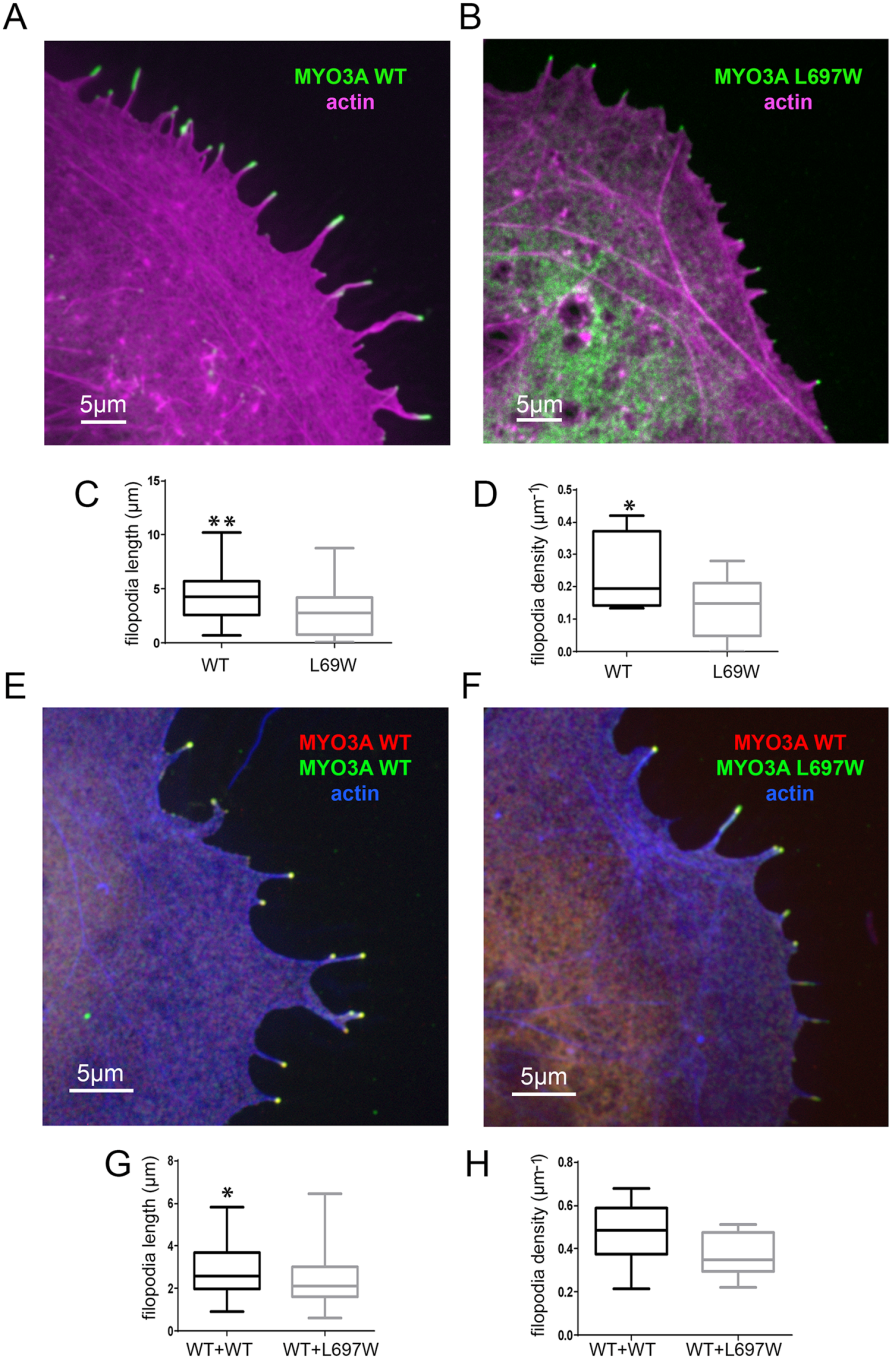
Impact of L697W mutation on MYO3A function. (**A**) Confocal images of transfected COS7 cells showing filopodia tip localization of GFP-MYO3A WT (green) or (**B**) GFP-MYO3A L697W (green). F-actin was stained with phalloidin and it is shown in magenta. (**C**) Filopodia length in WT and L697W coexpressing cells are presented as box plots, with upper and lower whiskers representing the maximum and minimum value, top, and bottom of the boxes representing the upper and lower 25th percentile and the bars bisecting the boxes representing the median values. P values were calculated using one-way ANOVA analysis are shown as asterisks (**p < 0.01 and *p < 0.05). (**D**) Filopodia initiation activity for WT and L697W measured as filopodia length and number per 10μm of cell perimeter and represented as in c. (**E**) GPFP-MYO3A WT and (**F**) L697W (green) retained tip localization when coexpressed with mCherry-MYO3A WT (red) in COS7 cells. F-actin was stained with phalloidin and it is shown in blue. (**G**,**H**) Filopodia length and initiation activity of WT and L697W in presence of mCherry-MYO3A WT (WT + WT and WT + L697W, respectively) represented as in (**C**,**D**).

When expressed in cultured cells, MYO3A has the ability to induce and elongate filopodial protrusions and localize to the filopodia tips^12,20,21^. These activities of MYO3A are proposed to be correlated to its motor activity^21,22^. We examined the filopodia initiation and elongation activity of the GFP tagged mutant MYO3AΔK L697W (MYO3A L697W) and mCherry tagged wild-type MYO3AΔK (MYO3A WT) constructs in COS7 cells. We observed that although MYO3A L697W showed filopodia tip localization similar to MYO3A WT (Fig. 4A, B), there was a significant reduction in MYO3A L697W filopodia initiation and elongation activities compared to MYO3A WT (p < 0.05 and p < 0.01, respectively) (Fig. 4C, D).

Due to the dominant nature of the L697W mutation observed in patients from the two families analyzed in this study, we decided to explore the potential influence of the mutant myosin on the wild-type when coexpressed in COS7 cells. We studied filopodia initiation and elongation in COS7 cells coexpressing MYO3A L697W and MYO3A WT. When coexpressed, both MYO3A WT and MYO3A L697W retained tip localization (Fig. 4E, F). However, there was a significant reduction in filopodia length (p < 0.05) between cells coexpressing GFP- MYO3A L697W and mCherry- MYO3A WT compared to cells coexpressing GFP- MYO3A WT and mCherry- MYO3A WT (Fig. 4G). Nevertheless, we did not observe a significant difference in filopodia initiation between these two conditions (Fig. 4H).

### Impact of L697W mutation on MYO3A filopodia initiation and elongation activity in COS7 cells in the presence of ESPN-1

**Figure 5:**
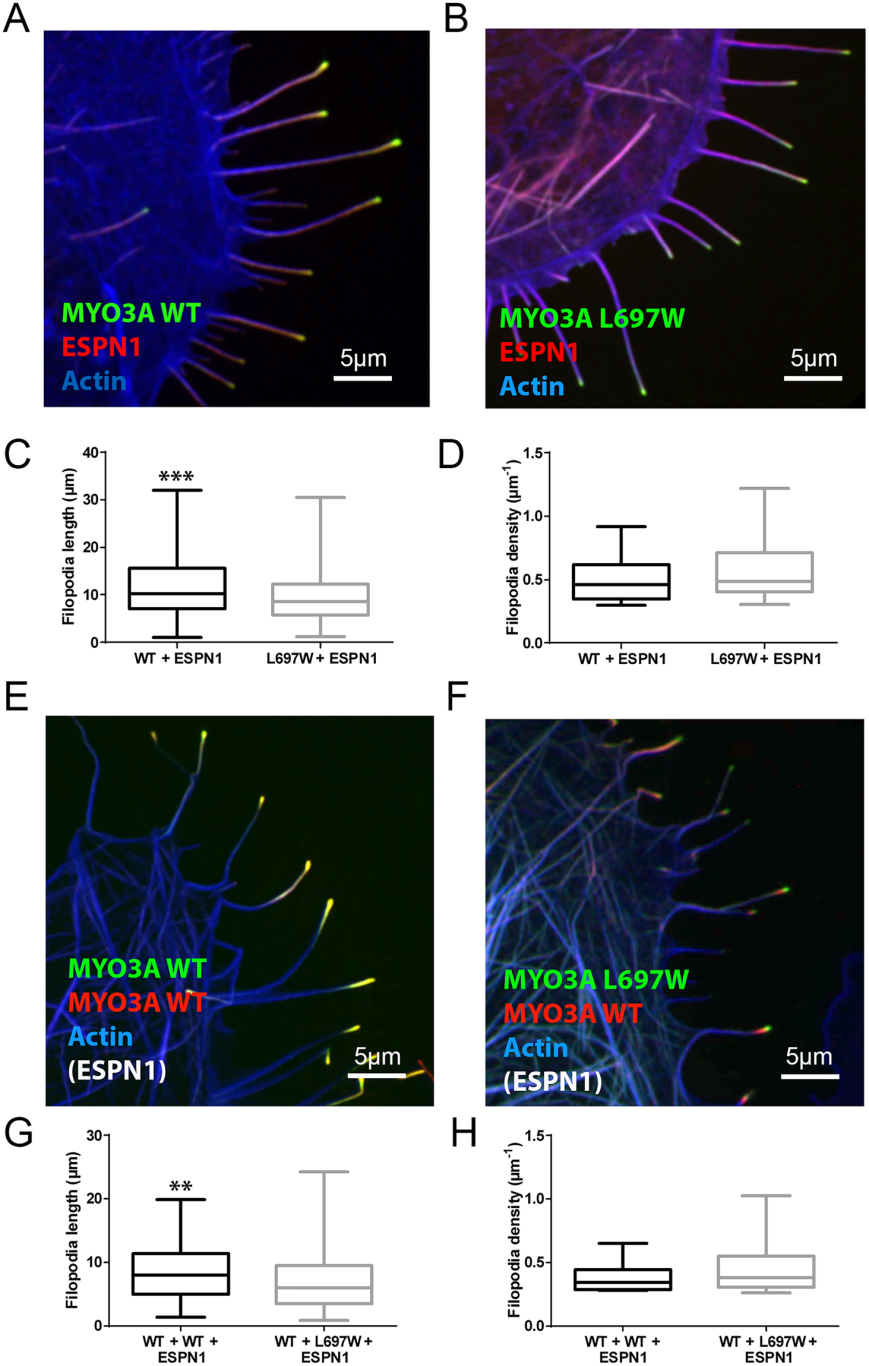
Impact of the L697W mutation on MYO3A function in the presence of ESPN-1. (**A**) Confocal images of transfected COS7 cells showing filopodia tip localization of GFP-MYO3A WT (green) or (**B**) GFP-MYO3A L697W (green) in presence of mCherry-ESPN-1 (red). (**C**,**D**) Filopodia length and initiation activity in WT and m-Cherry-ESPN-1 (WT + ESPN-1), and L697W and m-Cherry-ESPN-1 (L697W + ESPN-1) coexpressing cells are shown and represented as in Fig. 4. (**E**) Filopodia tip localization of WT (green) or L697W (**F**) when coexpressed with mCherry-MYO3A WT (red) and untagged ESPN-1 in transfected COS7 cells. (**G**,**H**) Filopodia length and initiation activity of transfected COS7 cells coexpressing WT, mCherry-MYO3A WT and untagged ESPN-1 (WT + WT + ESPN-1) and COS7 cells coexpressing L697W, mCherry-MYO3A WT and untagged ESPN-1 (L697W + WT + ESPN-1) represented as in Fig. 4. For all the confocal images, F-actin was stained with AlexaFluor®-405 phalloidin and it is shown in blue.

In order to examine the impact of the L697W mutation on MYO3A in the presence of ESPN-1, a known MYO3A cargo with actin regulatory properties^12^, we coexpressed MYO3A WT and MYO3A L697W constructs with ESPN-1 in COS7 cells and quantified average filopodia density and length. When coexpressed individually with N-terminal mCherry tagged ESPN-1 (ESPN-1), MYO3A L697W forms a comet-tail pattern of colocalization with ESPN-1 at filopodia tips similar to that previously reported for MYO3A WT^23^ (Fig. 5A, B). However, the filopodia length of MYO3A L697W and ESPN-1 coexpressing cells was significantly reduced compared to cells coexpressing MYO3A WT and ESPN-1 (p < 0.001) (Fig. 5C). We observed no significant difference in filopodia density of cells coexpressing MYO3A WT and ESPN-1 or MYO3A L697W and ESPN-1 (Fig. 5D).

To evaluate whether MYO3A L697W could exert a dominant negative effect on MYO3A WT in the presence of ESPN-1, we coexpressed both GFP-MYO3A L697W and mCherry-MYO3A WT with untagged ESPN-1 in COS7 cells. The coexpression of GFP-MYO3A L697W with mCherry-MYO3A WT and untagged ESPN-1 demonstrated a significant reduction of filopodia length compared to cells coexpressing GFP-MYO3A WT, mCherry-MYO3A WT and untagged ESPN-1 (p < 0.01) (Fig. 5E, G). We did not detect a significant difference in filopodia density between these two combinations (Fig. 5F, H). These data suggest that MYO3A L697W can exert dominant negative effect on the MYO3A WT role in actin protrusion elongation, but not in its role in actin protrusion initiation.

### Characterization of cargo specific tip localization behavior of MYO3A L697W

We have previously shown that myosins with different kinetic properties accumulate differentially at the tips of actin protrusions, where they compete for space in the local crowded molecular environment.^22,23^. For example, at steady state MYO3A can displace MYO3B at filopodia tips when they are both co-expressed in presence of the ESPN-1 cargo^23,24^. Our current data shows that MYO3A WT and MYO3A L697W in presence of ESPN-1 are enriched at the tips of filopodia forming the characteristic tip-to-base or “comet tail” gradients (Fig. 5E, F). We hypothesized that because of its unique kinetic properties, MYO3A L697W could exert a dominant negative effect on MYO3A WT function by competing and displacing MYO3A WT from its characteristic tipward accumulation in actin protrusions. We examined the tip localization profiles of mCherry-MYO3A WT and GFP-MYO3A L697W when coexpressed in the presence and absence of untagged ESPN-1.

**Figure 6:**
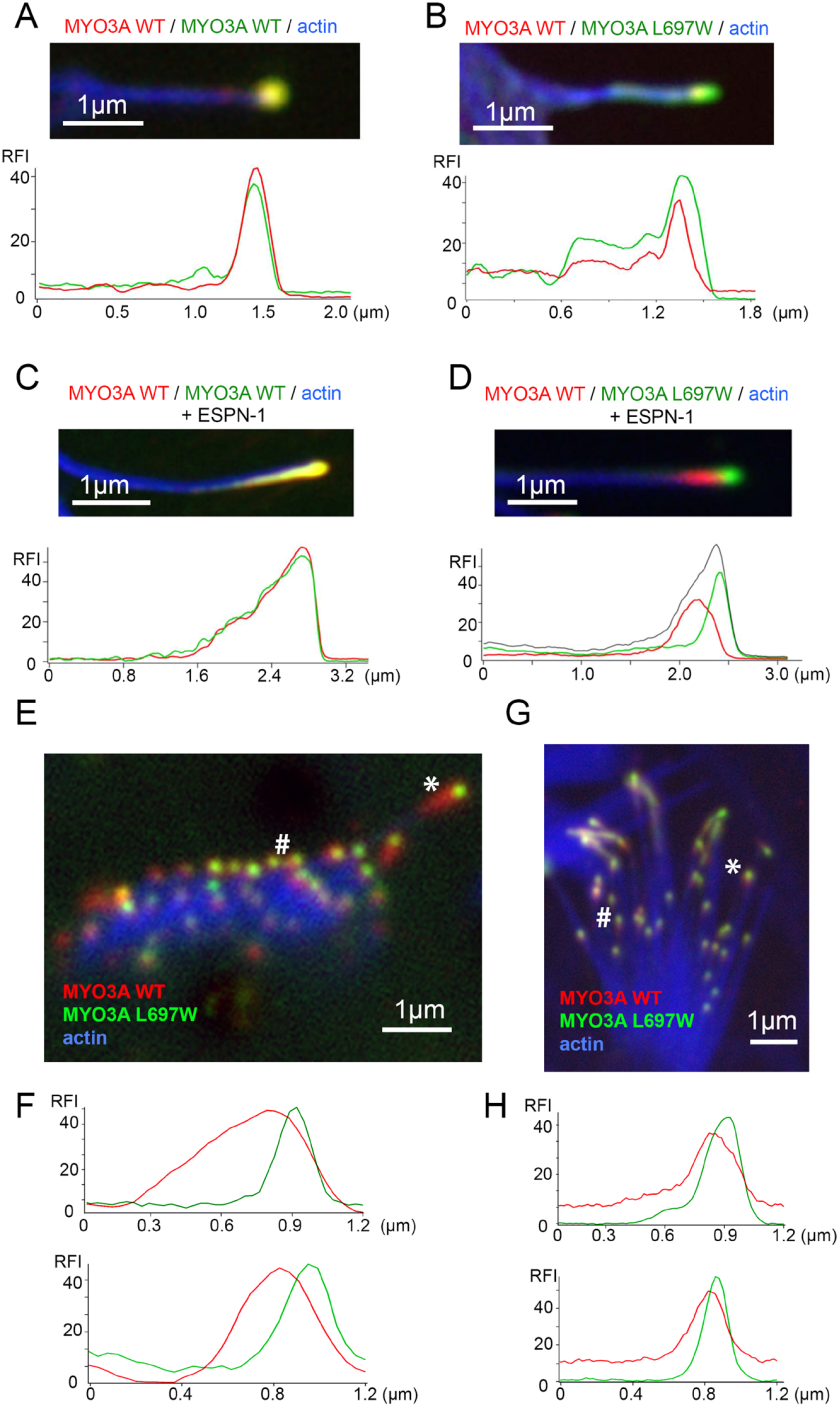
Cargo specific tip localization behavior of MYO3A L697W. (**A**) Close-up view of representative filopodia and fluorescence profiles along the filopodia length of COS7 cells coexpressing wild-type GFP-MYO3A WT (green) and mCherry-MYO3A WT (red). (**B**) Expression pattern and fluorescence profiles of mutant GFP-MYO3A L697W (green) and mCherry-MYO3A WT (red) proteins in a representative COS7 cell filopodia (**C**) Filopodia localization and fluorescence profiles of GFP-MYO3A WT (green), mCherry-MYO3A WT (red) proteins in a COS7 cell coexpressing GFP-MYO3A WT, mCherry-MYO3A WT and untagged ESPN-1. (**D**) Close-up view of filopodia and fluorescence profiles along it of COS7 cells coexpressing GFP-MYO3A L697W (green), mCherry-MYO3A WT (red), actin (in blue) and untagged ESPN-1. The sum of the green and red channels (black trace in the fluoresce profile) shows the same profile as MYO3A WT in the previous panel. (**E**–**G**) Confocal images of transfected mouse auditory (**E**) and vestibular (**G**) hair cells coexpressing mCherry-MYO3A WT (red) and GFP-MYO3A L697W (green) proteins. (**F**–**H**) Fluorescence profiles along the stereocilia length of auditory (**F**) and vestibular (**H**) hair cells coexpressing mCherry-MYO3AWT (red) and GFP-MYO3A L697W (green). For all the images, actin was stained with AlexaFluor®-405 phalloidin and it is shown in blue.

In absence of ESPN-1, GFP-MYO3AWT and mCherry-MYO3AWT coexpression in COS7 cells showed overlapping fluorescence profiles with a steep tip-to-base gradient at the filopodia tips (Fig. 6A). Coexpression of MYO3A WT and MYO3A L697W also demonstrated a steep tip-to-base gradient but MYO3A L697W exhibited a slightly more tipward position compared to MYO3A WT (Fig. 6B). Similarly, in presence of ESPN-1, coexpression of GFP-MYO3A WT and mCherry-MYO3A WT showed a robust overlap of the fluorescence profiles at the filopodia tips for both WT-tagged proteins (Fig. 6C). Whereas coexpression of GFP-MYO3A L697W and mCherry-MYO3A WT in the presence of ESPN-1 demonstrated a predominant tipward localization of MYO3A L697W with MYO3A WT trailing behind with a relatively shallower tip-to-base gradient (Fig. 6D). Interestingly, the sum of the MYO3A WT and L697W fluorescent intensity profiles (grey line, Fig. 6D) shows a tip to base comet tail decay similar to that observed when expressing GFP- MYO3A WT or mCherry-MYO3A WT, indicating that the overall distribution of MYO3A is not affected when the combination of both WT and L697W mutant forms are present. These data suggest that MYO3A L697W displaces MYO3A WT from the tips of actin protrusions and that this effect is enhanced in the presence of ESPN-1 cargo. Our actin binding data combined with cellular localization observations suggests that this could be a result of MYO3A L697W presenting a higher stability (residency time) at the tips. These observations support our hypothesis that MYO3A L697W can exert dominant negative effect on MYO3A WT.

### Characterization of the impact of L697W mutation on MYO3A localization in hair cells

MYO3A is known to localize at the tips of vertebrate hair cell stereocilia in a steep tip-to-base gradient^12,13^. The differing tip localization pattern of MYO3A WT and MYO3A L697W when coexpressed in the presence and absence of ESPN-1 in COS7 cells prompted us to examine their localization pattern in hair cell stereocilia. Exogenous coexpression of mCherry-MYO3A WT and GFP-MYO3A L697W in mouse cochlear explants demonstrated a consistent tipward localization of MYO3A L697W and proximal localization of MYO3A WT in both auditory and vestibular hair cell stereocilia (Fig. 6E–H). This localization pattern was similar to the localization pattern we observed in COS7 cells coexpressing MYO3A WT, MYO3A L697W and ESPN-1 (Fig. 6D), confirming that MYO3A L697W can exert a dominant negative effect by displacing the MYO3A WT protein from the tips of stereocilia.

## Discussion

Walsh *et al*.^3^ investigated a three-generation Jewish family with individuals presenting an onset of hearing loss near the second decade of life and affected by autosomal recessive nonsyndromic hearing loss. Three different mutations were identified in *MYO3A* (DFNB30) in this study and all the affected individuals carried at least two of these mutations, some of them being homozygotes and others being compound heterozygotes for the mutations^3^. Since then, several reports have identified additional *MYO3A* mutations associated with autosomal recessive hearing loss.

The variant described in the current study has not been reported in any of the mutation databases (1000 Genomes Project, 6500 Exomes, and ExAC), and prediction programs indicated it as likely pathogenic. Our genetic and clinical data from two unrelated families clearly show that the mutation segregates with autosomal dominant hearing loss. The *MYO3A* variant c.2090 T > G identified in this work leads to a substitution of amino acid 697 in MYO3A motor domain, replacing leucine with tryptophan. The leucine 697 is located in the U50 domain of MYO3A motor, exposed to the solvent and far from the ATP-binding pocket and the actin-binding domain. However, when we introduced the mutation in the predicted MYO3A structure (Fig. 3A,B), we observed a clash between tryptophan 697 and lysine 462 that may disrupt the conformational changes in the U50 domain. Spectroscopic and crystallographic studies have demonstrated that conformational changes in the U50 domain are coordinated with the movement of the conserved switch I region of the active site, which is critical for coordinating nucleotide binding and hydrolysis^25–28^. Since the U50 domain also interacts with actin, its conformation is altered by actin binding and the conformation of switch I in the active site and vice-versa. Our biochemical and biophysical data suggests that the mutation could impair the actin-activation of ADP release and slow the ADP release rate constant. The slower ADP release rate constant would be consistent with the slower ATPase and *in vitro* motility of MYO3A L697W we measured, suggesting that the mutant has an increase in duty ratio (fraction of the ATPase cycle that myosin motor is bound to actin). Indeed direct measurements of steady-state actin affinity (K_Actin_) and the actin-dependence of the ATPase assay (K_ATPase_) indicate that the L697W mutant enhances duty ratio and overall actin affinity of MYO3A. Interestingly, leucine 697 is conserved in all MYO3 family members, but it is not conserved in other myosins. The observation that a single amino acid change in this particular location of the MYO3A motor domain affects protein function may help elucidate the unique structure-function mechanisms proposed for this unique motor (e.g. proposed MYO3 inchworm motility mechanism^24^).

In our previous study, we proposed that tip localized MYO3A functions as a motorized actin crosslinker, a property which would require an active motor domain and intact tail domain^21^. Our previous results demonstrated that MYO3A lacking an intact tail domain retains tip localization but fails to induce or elongate actin protrusions^21^. Our current results demonstrate that MYO3A L697W with reduced motor activity also retains a tip localization phenotype, however, shows a significantly reduced ability to induce and elongate actin protrusions (Fig. 4). Altogether, these results strongly suggest that MYO3A requires an active motor domain and intact tail domain for tip localization as well as actin protrusion initiation and elongation activity. Our results are consistent with previously published data and we predict that the reduced actin protrusion elongation activity of MYO3A L697W is correlative to its reduced motor activity^20,22^. It is important to note that although the MYO3A L697W mutant is able to tip localize in actin based protrusions (filopodia and stereocilia), the members of the two pedigrees carrying the mutation developed deafness. We believe that our results demonstrating reduced actin protrusion initiation and elongation activity of tip localized MYO3A L697W in the absence or presence of ESPN-1 (Figs. 4A–D and 5A–D), partially explains the dominant negative impact of this mutant form. Furthermore, coexpression of MYO3A WT with MYO3A L697W in the presence or absence of ESPN-1 rescued filopodia initiation but not filopodia elongation activity (Figs. 4E–H and 5E–H), demonstrating the dominant negative effect of the mutant form on actin protrusion elongation.

It has been proposed that the ability of MYO3A to transport ESPN-1, ESPNL and Protocadherin-15 along parallel actin bundle-based protrusions is crucial for maintaining the ultrastructure and function of the vertebrate inner ear hair cell stereocilia^12,13,16,29^. The specificity of interactions between MYO3 isoforms and its proposed cargo leads to differential regulation of stereocilia length^13^. For example, ESPNL has been shown to facilitate shorter actin protrusions in the presence of MYO3A and longer actin protrusions in the presence of MYO3B^13^. MYO3A and MYO3B coexpression in the presence of ESPN-1 lead to a differential tip localization pattern, such that MYO3A WT predominantly occupies a tipward position, while MYO3B localizes proximally^23^. It is believed that the combination of higher motor activity and actin binding affinity of MYO3A compared to MYO3B is a major cause for the observed distribution pattern^23,24^. In this study, we observed a difference in the fluorescence profiles of MYO3A WT and MYO3A L697W when coexpressed in the presence or absence of ESPN-1 (Fig. 6A–D). It is important to note that there is an enhanced distribution of MYO3A WT and MYO3A L697W fluorescence peaks at actin protrusion tips in the presence of ESPN-1 compared to their distribution in the absence of ESPN-1 (Fig. 6). The enhanced distribution of MYO3A WT and MYO3A L697W fluorescence peaks in the presence of ESPN-1 highlights the complexity of MYO3A regulation in the presence of its multiple cargoes within the vertebrate hair cell stereocilia.

We speculate that the enhanced duty ratio of MYO3A L697W may allow it to be retained at the distal actin protrusion tips more efficiently than MYO3A WT. The observed inversely proportional amounts of MYO3A WT and MYO3A L697W at the overlapping regions near the filopodia tips suggest that the two proteins may be competing to occupy a distal (tipward) position. Our results are suggestive of a mechanism where MYO3A L697W is capable of transporting ESPN isoforms from the base to the tips of stereocilia, however, it may not be able to influence the ESPN turnover rate similar to MYO3A WT, which may gradually lead to stereocilia degeneration. It is also possible that MYO3A L697W is unable to maintain the turnover and function of Protocadherin-15^16^, a crucial component of the mechanotransduction complex or inner hair cells essential for hearing. Overall, these results suggest that although MYO3A L697W could compete with MYO3A WT and retain tip localization phenotype, MYO3A L697W cannot compensate for retaining normal actin regulation and cargo transport functionality. The predominant tipward localization pattern demonstrated by MYO3A L697W in both filopodia and stereocilia strengthens our hypothesis that the dominant tipward localization phenotype may be a major factor that contributes to the gradual loss of hearing function. The gradual hearing loss phenotype highlights that MYO3A and its cargoes are crucial for maintaining stereocilia length and function, but also demonstrates that compensatory mechanisms for lack of function mutations in MYO3A can be quite complex.

Interestingly, the variant was found in two unaffected individuals from family 1 (Fig. 1, VI-18 and VI-22) who are young (28 and 29 years old), below the maximum age of onset of hearing loss observed in other family members, and in one individual (30 years old) from family 2 (Fig. S1, IV-11). We speculate that they are non-penetrant or late-onset carriers. It is also possible that MYO3B could partially compensate for the effects of mutations in MYO3A and the effectiveness of this compensation could vary among individuals, explaining non-penetrance and differences in age of onset of hearing loss. MYO3B has a slightly different spatiotemporal expression pattern than MYO3A^23,24^ and the possibilities for its involvement in the delay of the onset of hearing loss in individuals with the dominant MYO3A L697W mutation are complex and difficult to predict. The presence of congenital hearing loss in individual V:1, from family 2 is also noteworthy. Hearing loss was detected at birth after neonatal screening and the child underwent cochlear implantation at the age of 2. Frequent mutations associated with hearing loss were excluded in this case (Sanger sequencing of *GJB2* gene, screening of deletions in *GJB6* gene and m.1555 A > G mutation were performed). The contribution of either environmental or genetic factors in causing early onset of hearing loss cannot be ruled out.

The first known deafness causing MYO3A dominant mutation (MYO3A G488E) was reported recently in a small African American family^16^. The G488E mutation is located near the switch I region of the MYO3A motor domain and reduces the ATPase activity and increases *in vitro* actin sliding velocity compared to MYO3A WT^16^. It was also demonstrated that MYO3A G488E was capable of reaching the tips of rat organotypic inner ear culture stereocilia, and showed a localization pattern similar to that seen for wild-type MYO3A^16^. However, MYO3A G488E failed to tip localize in COS7 cell filopodia. Thus, although both L697W and G488E mutations are associated with dominant phenotypes, their impact on the structure and/or function of the MYO3A motor is distinct. It will be interesting to further examine the impact of these mutations on the kinetic steps of MYO3A ATPase cycle and their differential impact on cellular functions. Overall, our results highlight that MYO3A mutations leading to subtle changes in biochemical properties appear to have a major impact on stereocilia initiation and ultrastructure maintenance. Our results suggest that a combination of 1) complexity of MYO3- multiple cargo system, 2) competition and compensation of the mutant MYO3A with MYO3A WT and MYO3B WT, and 3) proposed redundant function of MYO3B WT in the absence of MYO3A, may contribute to the observed variation in the age of hearing loss onset and extent of progression.

## Materials and Methods

### Patients

A seven-generation family (Family 1; Fig. 1), from the Southeastern region of Brazil, was ascertained for molecular studies and 52 DNA samples were collected: 27 samples were collected from affected individuals and 25 belong to unaffected ones. From a second family (Family 2; Fig. S1a), also from the Southeastern region of Brazil, 21 samples were collected: 11 from affected individuals and 10 from unaffected. In both families, autosomal dominant transmission of hearing loss was observed. The two families were apparently unrelated. Written informed consent was obtained from participants or guardians of participants. The study was approved by the Institutional Ethics Committee (Biosciences Institute, University of São Paulo).

### Audiological evaluation

In general, affected and unaffected individuals underwent otological and audiologic evaluation. Pure tone audiometry, both air (frequencies ranging from 250 to 8000 Hz) and bone conduction (frequencies ranging from 500 to 4000 Hz) were performed to identify hearing threshold levels. Vocal audiometry included speech reception threshold (SRT) and speech recognition index. We also performed acoustic immittance measurements, including tympanometry and acoustic reflexes thresholds.

### DNA extraction

Blood samples were collected and DNA extraction was conducted with the help of Autopure LS Equipment (Gentra Systems, Minneapolis, Minnesota, USA) in samples from family 1. From samples of family, DNA was extracted with Easy-DNATM Kit (Version D) Genomic DNA Isolation” from Invitrogen (Thermo Scientific, Waltham, MA, USA) and GFX Genomic Blood DNA Purification Kit” from Amershan Biosciences (GE Healthcare Life Sciences, Amersham, Buckinghamshire, UK).

### Whole-exome sequencing

DNA samples from four affected individuals of family 1 (proband V:4; V:15, V:24, and V:34) were submitted to whole-exome sequencing at Laboratório de Biotecnologia Animal (ESALQ-USP, Piracicaba, SP, BR). The library was prepared with an Illumina TruSeq library preparation kit. Sequence capture was performed with a target Illumina Exome enrichment kit (~62 Mb size). Sequencing was performed with an Illumina HiSeq. 2000 (Illumina INC, San Diego, California, USA) and the average read depths were 120×. Fastq files were aligned to the human reference sequence (hg19) with Burrows-Wheeler Aligner (BWA)^30^ for the generation of the SAM files. SAM to BAM conversion and BAM files sorting and PCR duplicates marking was performed with Picard (http://picard.sourceforge.net) Realignment of indel regions, variant detection and recalibration of base qualities were performed with Genome Analysis Tool Kit (GATK)^31^ for the production of VCF files. Annotation was performed with Annovar^32^. Variant frequencies were compared to the 1000 Genomes Project (http://www.1000genomes.org), ESP6500 (http://evs.gs.washington.edu/EVS/), 65000 exomes from the Exome Aggregation Consortium (ExAC) (http://exac.broadinstitute.org/) and the Brazilian ABraOM (http://abraom.ib.usp.br) databanks. PolyPhen2^33^, SIFT^34^ and Mutation Taster^35^ were used for *in silico* damage prediction to the protein. A similar method was used for sequencing DNA samples from four affected individuals of family 2 (proband III-22, III-3, III-13, IV-8). Whole-exome sequencing was performed at Universidade de Campinas (UNICAMP, SP, BR).

### Sanger Sequencing

Primers for amplification of the exons containing the candidate variants were designed with Primer3 (http://bioinfo.ut.ee/primer3-0.4.0/). PCR fragments were purified and directly sequenced in both strands using the ABI BigDye Terminator v3.1 Cycle Sequencing Kit and the ABI 3730 DNA Analyzer (Applied Biosystems, Foster City, CA, USA). Primer sequences used were: *MYO3A-F* (forward) 5′-TTCATTTTTGGGGAGTGACC-3′ and *MYO3A-R* (reverse) 5′-GTAGACTTACATCACCTGACATTTGG-3′; *LONP2-F* (forward) 5′- TGAACCTGAGAGGTGGAGGT-3′ and *LONP2-R* (reverse) 5′- CTCAGTAAATACTCAAAAGTTGCCTG-3′.

### Linkage analysis

Two-point lod scores were calculated with Merlin^36^, one point being the hearing loss phenotype and the other the presence of the novel variant; the allele frequency was set at 0.0001. The penetrance was assumed to be complete.

### Array-CGH

Array-CGH was performed in DNA samples from one affected patient from each of the two families using a platform from Agilent Technologies containing 180.000 oligonucleotides, as described by the manufacturer. Data were processed with Feature Extraction software and subsequently analyzed with the Genomic Workbench software (Agilent Technologies, Santa Clara, CA, USA). Gains and losses of genomic sequences were called using the aberration detection statistical algorithm ADM-2, with a sensitivity threshold of 6.7.

### SNP-array genotyping and Kinship analyses

The samples were genotyped with the high-density SNP array - Axiom Human Origins (~ 600 K SNPs - Affymetrix/ThermoFisher). Data cleaning and filtering were performed as described^37^. In order to evaluate the relation of kinship between the individuals of the different families we estimated the IBD using the moments method implemented in SNPRelated R package^38^.

### Protein expression and purification

The Baculovirus SF9 insect cell system was used to express recombinant MYO3AΔK 2IQ c-GFP WT and MYO3AΔK 2IQ c-GFP L697W with a N-terminal FLAG tag and purified with anti-FLAG affinity chromatography as described previously^20,24,39^.

### Myosin ATPase Assay

The steady state enzyme-linked ATPase assay was used to examine MYO3AΔK 2IQ WT c-GFP and MYO3AΔK 2IQ L697W c-GFP actin-activated ATPase activity in KMg50 buffer (50 mM KCl, 1 mM EGTA, 1 mM MgCl_2_, 1 mM ATP, 10 mM Imidazole pH 7.0, 1 mM DTT) at 25 °C^9,20,21,39^. Briefly, the mutant and WT MYO3A motor ATPase was examined in the presence of a range of actin concentrations in an Applied Photophysics stopped-flow. The Michaelis-Menten equation was used to determine the K_ATPase_ (actin concentration at which the ATPase activity is one-half maximal) and k_cat_ (maximal actin-activated ATPase rate), using a hyperbolic fit of the ATPase rates as a function of actin concentration.

### Actin Co-sedimentation Assay

The steady-state actin affinity of MYO3AΔK 2IQ c-GFP in the presence of ATP was measured using an actin co-sedimentation assay. MYO3A (0.5 µM) was equilibrated with various actin concentrations (0–60 µM) in KMg50 buffer containing the ATP regeneration system (20 unit s^−1^·ml^−1^ pyruvate kinase and 2.5 mM phosphoenolpyruvate). MYO3A was pre-spun for 10 min at 4 °C (TLA.120.1 rotor at 95,000 rpm) prior to equilibrating it with actin. We added 1 mM ATP and immediately centrifuged the samples for 10 min at 25 °C (TLA.120.1 rotor at 95,000 rpm). The supernatant was examined by GFP fluorescence using a 96 well plate reader (ex:485 nm/em: 530 nm long pass). The fraction bound was determined by examining the relative decrease in GFP fluorescence in the supernatant compared to the sample without actin [1 − (sup/0 actin sup)]. The supernatant and pellet were also examined by SDS-PAGE followed by coomassie staining to detect MYO3A. The intensity of the MYO3A bands was examined using ImageJ and the fraction bound was calculated [(pellet/(supernatant + pellet)]. The plot of fraction bound as a function of actin concentration was fit to a hyperbolic function to determine the steady-state actin affinity (K_actin_). Both the coomassie staining and GFP fluorescence methods yielded similar results.

### *In Vitro* Motility Assay

The *in vitro* motility assay^40^ was used to determine the actin filament motility of MYO3AΔK 2IQ WT c-GFP and MYO3AΔK 2IQ L697W c-GFP as described previously^21,40,41^. Briefly, the nitrocellulose-coated glass coverslip surface was coated with anti-GFP antibody (Life Technologies). The surface of the coverslip was then blocked with 1 mg ml^−1^ BSA solution in KMg50 buffer, followed by addition of C-terminal GFP tagged MYO3AΔK 2IQ WT or MYO3AΔK 2IQ L697W. Rhodamine labelled F-actin was then added to the flow chamber which was followed by the addition of activation buffer, which consisted of KMg50 supplemented with 0.35% methylcellulose, 1 mM DTT, 10 µM calmodulin, 1 mg ml^−1^ BSA, 2 mM ATP and an ATP regeneration system (20 units ml^−1^ pyruvate kinase, 2.5 mM phosphoenolpyruvate). To reduce photobleaching 1 mg ml^−1^ glucose, 0.1 mg ml^−1^ glucose oxidase and catalase were included in the activation buffer. After the addition of activation buffer, the motility of the rhodamine-phalloidin labeled F-actin filaments was observed using Nikon TE2000 microscope^41^. The time-lapse images were acquired at 5–10 s intervals for a period of 10–15 minutes. The velocity of moving actin filaments was measured using ImageJ with MtrackJ plugin^42^. A mixture assay was used to examine if the presence of the mutant L697W MYO3A could slow down WT MYO3A motility. The total amount of MYO3A was held constant while varying ratios of pre-mixed mutant/WT were loaded into the flow cell and the average velocity in each condition determined.

### MYO3A motor domain homology modeling

A homology model of the human MYO3A motor domain (amino acids 338 to 1053 of NM_017433.4), was generated using SWISS-MODEL^19^ and crystal structure of the human MYO10 on the pre-power stroke state (PDB ID: 5i0h) as a template. To do so, we first generated a hidden Markov model (HMM)-based sequence profile containing MYO3A homologs using the HHblits server^43^. This HMM-based sequence profile was used to search for the best template in a database (pdb70) containing all the structurally resolved proteins using the HHpred server^44^. Several valid templates presenting similar sequence coverage and identity were identified, and the 5 candidates presenting higher scores (PDB IDs: 5m05, 1w9i, 4qbd, 5ioh, and 4zg4) were used to generate a total of 5 MYO3A homology models using SWISS-MODEL^19^. Residues 873–924 were not conserved or absent in other myosins and were removed from the models. The 5 models were first refined with 3Drefine^45^ and later analyzed with PROCHECK^46^. The model based on MYO10 (PDB ID: 5i0h) presented the best Ramanchandran plot as assesed by PROCHECK^46^ and had the highest sequence identity (39.2%) to the modeled region of MYO3A, and was therefore selected for further analysis. The global root-mean-square deviation between the model and template was 2.16 Å for 638 alpha carbons as estimated by SuperPose V1.0^47^ and 92.8% of residues were in the most favored regions of the Ramachandran plot^46^. The ADP molecule and magnesium ion from the template structure were superposed on the MYO3A model to indicate the conserved ATP binding site. Leucine 697 was mutated to tryptophan using UCSF Chimera^48^ and the most common tryptophan rotamer (0.2687 probability) from the Dunbrack library is shown. The final figure was generated in UCSF Chimera^48^ and ray-traced images were produced with POV-ray (http://www.povray.org/).

Protein sequence alignment showing L697 residue conservation among human myosin proteins was generated with Clustal Omega^49^ using human myosin sequences obtained from Uniprot database and edited in Jalview^50^. Sequence alignment was colored based on the BLOSUM62 score with a conservation index of 20.

MYO3 protein sequences shown in Fig. S2 were identified by running a nucleotide BLAST^51^ search using the human MYO3A motor domain sequence and aligned with Clustal Omega^49^. To decrease redundancy, isoforms and uncharacterized proteins sequences were removed from the final alignment. A total of 45 sequences were shown in the final alignment including some MYO3B. Sequence alignment was edited in Jalview^50^ and colored based on sequence identity.

### Confocal microscopy analysis

COS7 cells (ATCC, CRL-1651) were trypsinized, plated on coverslips and maintained in Dulbecco’s Modified Eagle Medium (DMEM) supplemented with 10% fetal bovine serum (FBS) at 37 °C and 5% CO_2_ in a cell incubator. Cells were transfected using Lipofectamine transfection reagent (Invitrogen) per manufacturer’s instructions and incubated for 24 h for protein expression. Samples were then fixed for 20 min in 4% paraformaldehyde in Phosphate Buffered Saline (PBS), permeabilized and counterstained for actin with AlexaFluor®-405 phalloidin for 30 min in 0.5% Triton X-100 in PBS. Finally, samples were mounted on glass slides and imaged in a Nikon microscope equipped with a Yokogawa spinning disk confocal unit. ImageJ^52^ software was used to quantify the length and filopodia density (number of filopodia per 10 µm of cell perimeter), and to estimate the relative pixel intensity of fluorescently tagged proteins along filopodia. GraphPad Prism v6 software was used to perform the statistical analysis and generate the final graphs. Filopodia length and filopodia density data were presented as box plots, with upper and lower whiskers representing the maximum and minimum value, top and bottom of the boxes representing the upper and lower 25th percentile, and the bars bisecting the boxes representing the median values. P values were calculated using one-way ANOVA analysis and are represented with asterisks in the figures; * for 0.05 < p > 0.01, ** for 0.01 < p > 0.001 and *** for p < 0.001. A p < 0.05 was considered significant. Values from approximately 20 COS7 cells were used for each combination and the data from at least three independent experiments were included.

### Biolistic transfection of rat inner-ear tissue

The care and use of mice used for transfection experiments conformed to NIH guidelines, and were approved by the Institutional Animal Care and Use Committee of the National Institute on Deafness and Other Communication Disorders, NIH (protocol #1215). Inner-ear sensory epithelium (organ of Corti and vestibular organs) cultures were dissected from postnatal days 1-3 mouse and cultured on collagen-coated coverslips. Explants were bathed in DMEM/F12 (Invitrogen) supplemented with 10% FBS and 5 µg/mL ampicillin, and incubated overnight at 37 °C with 5% CO_2_. Tissue explants were then transfected with DNA-coated gold particles using a Helios Gene Gun System (Bio-Rad). Gold particles (1.0 μm, Bio-Rad) were coated with plasmid DNA at a ratio of 25 μg of gold particles to 50 μg of cDNA encoding mCherry-MYO3AΔK WT and GFP-MYO3AΔK L697W. DNA-coated particles were deposited onto the inner wall of Tefzel tubing (Bio-Rad), which was cut into individual cartridges. After transfection, samples were incubated in fresh supplemented DMEM/F12 media at 37 °C for 14–24 hours for protein expression. Tissue was them fixed with 4% paraformaldehyde in PBS and permeabilized and counterstained with AlexaFuor®-405 phalloidin for 30 min in 0.5% Triton X-100 in PBS to visualize stereocilia. Finally, samples were mounted on a glass slide and imaged as described before^13^.

### Data Availability

The datasets generated and/or analyzed during the current study are available from the corresponding author on reasonable request.

## Electronic supplementary material

Supplementary information
