# Supplementary material for: Characterization of a novel MYO3A missense mutation associated with a dominant form of late onset hearing loss
Source: Sci Rep. 2018 Jun 7;8:8706. doi: 10.1038/s41598-018-26818-2 (PMC5992146; doi:10.1038/s41598-018-26818-2)
Supplement: Supplementary file 1 — Supplementary information [file 41598_2018_26818_MOESM1_ESM.pdf]

SUPPLEMENTARY INFORMATION

Characterization of a novel *MYO3A* missense mutation associated with a dominant form of late onset hearing loss

Vitor G L Dantas[1¥], Manmeet H. Raval[2¥], Angela Ballesteros[3¥], Runjia Cui[3], Laura K. Gunther[2] Guilherme L Yamamoto[1], Leandro Ucela Alves[1], André Silva Bueno[1], Karina Lezirovitz[1,4], Sulene Pirana[5,6], Beatriz C.A. Mendes[7], Christopher M. Yengo[2*], Bechara Kachar[3*], Regina C Mingroni-Netto[1*].

1. Centro de Pesquisas sobre o Genoma Humano e Células-Tronco, Departamento de Genética e Biologia Evolutiva, Instituto de Biociências, Universidade de São Paulo, São Paulo, Brazil
2. Department of Cellular and Molecular Physiology, Pennsylvania State University, College of Medicine, Hershey, PA 17033, USA
3. Laboratory of Cell Structure and Dynamics, National Institute on Deafness and Other Communication Disorders, National Institutes of Health, Bethesda, MD 20892, USA
4. Laboratório de Otorrinolaringologia/LIM32 – Hospital das Clínicas, Faculdade de Medicina, Universidade de São Paulo, São Paulo, Brazil
5. UNIFAL - Universidade Federal de Alfenas, Alfenas, Brazil
6. Universidade São Francisco, Bragança Paulista, Brazil
7. Divisão de Educação e Reabilitação dos Distúrbios da Comunicação da Pontifícia Universidade Católica de São Paulo, São Paulo, Brazil

[¥] V.G.L.D., M.H.R. and A.B. contributed equally to this work.

*CORRESPONDING AUTHORS:

Regina Célia Mingroni Netto, Departamento de Genética e Biologia Evolutiva, Instituto de Biociências, Universidade de São Paulo, São Paulo, Brazil. Email: renetto@ib.usp.br

Christopher Yengo, Department of Cellular and Molecular Physiology, Pennsylvania State University, College of Medicine, Hershey, PA 17033, USA. Email: cyengo@pennstatehealth.psu.edu

Bechara Kachar, Laboratory of Cell Structure and Dynamics, NIDCD, NIH, Bethesda, MD 20892, USA Email: kacharb@nidcd.nih.gov

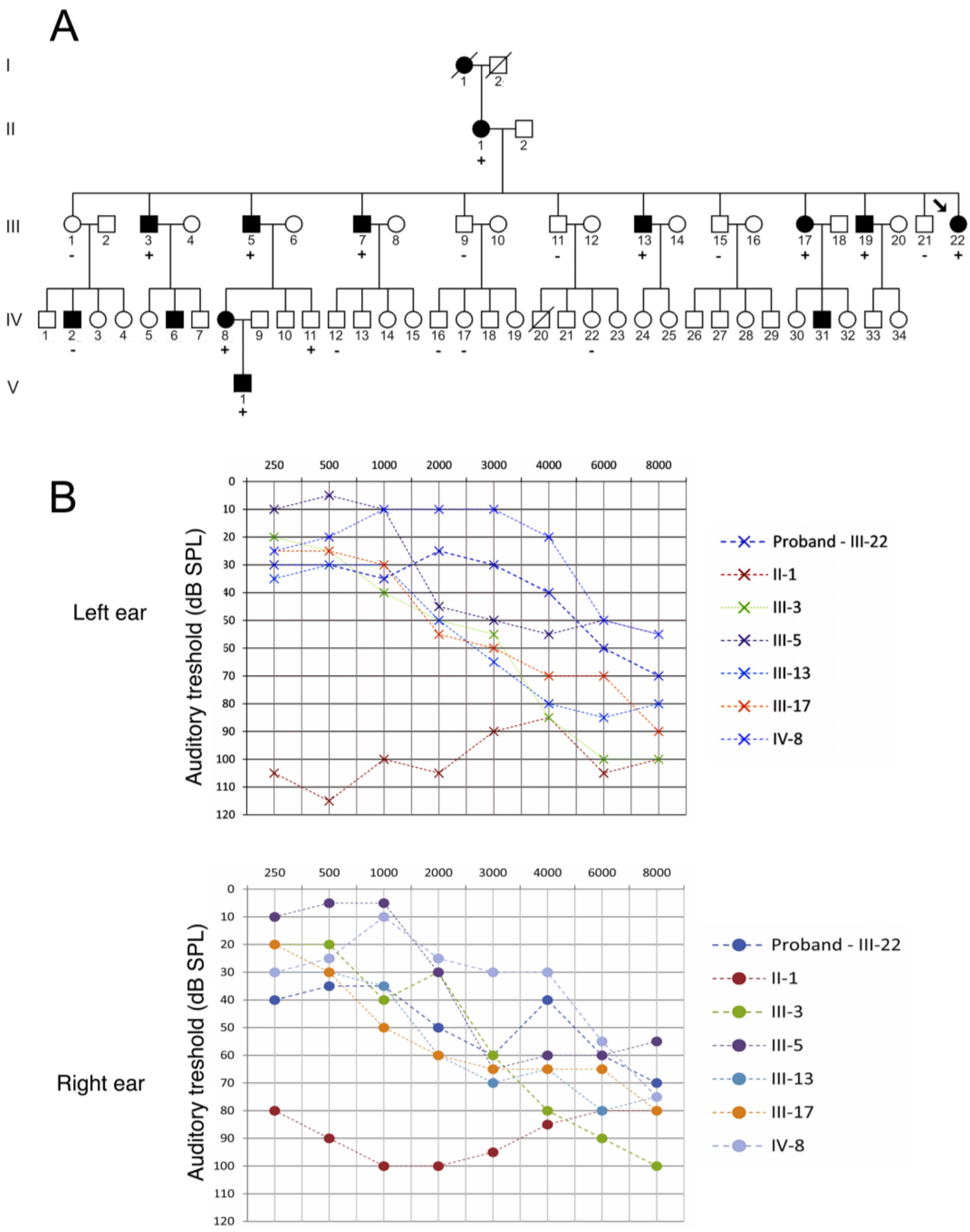

Figure S1: Family 2 pedigree and audiometry thresholds. (a) Pedigree of family 2 showing individuals whose samples were tested for the presence of the c.T2090G mutation in MYO3A. Individuals indicated with a plus sign (+) are heterozygotes for the c.T2090G mutation and individuals indicated with a minus sign (-) do not carry the mutation. (b) Left and right ear tonal audiometry thresholds of seven affected individuals from family 2.

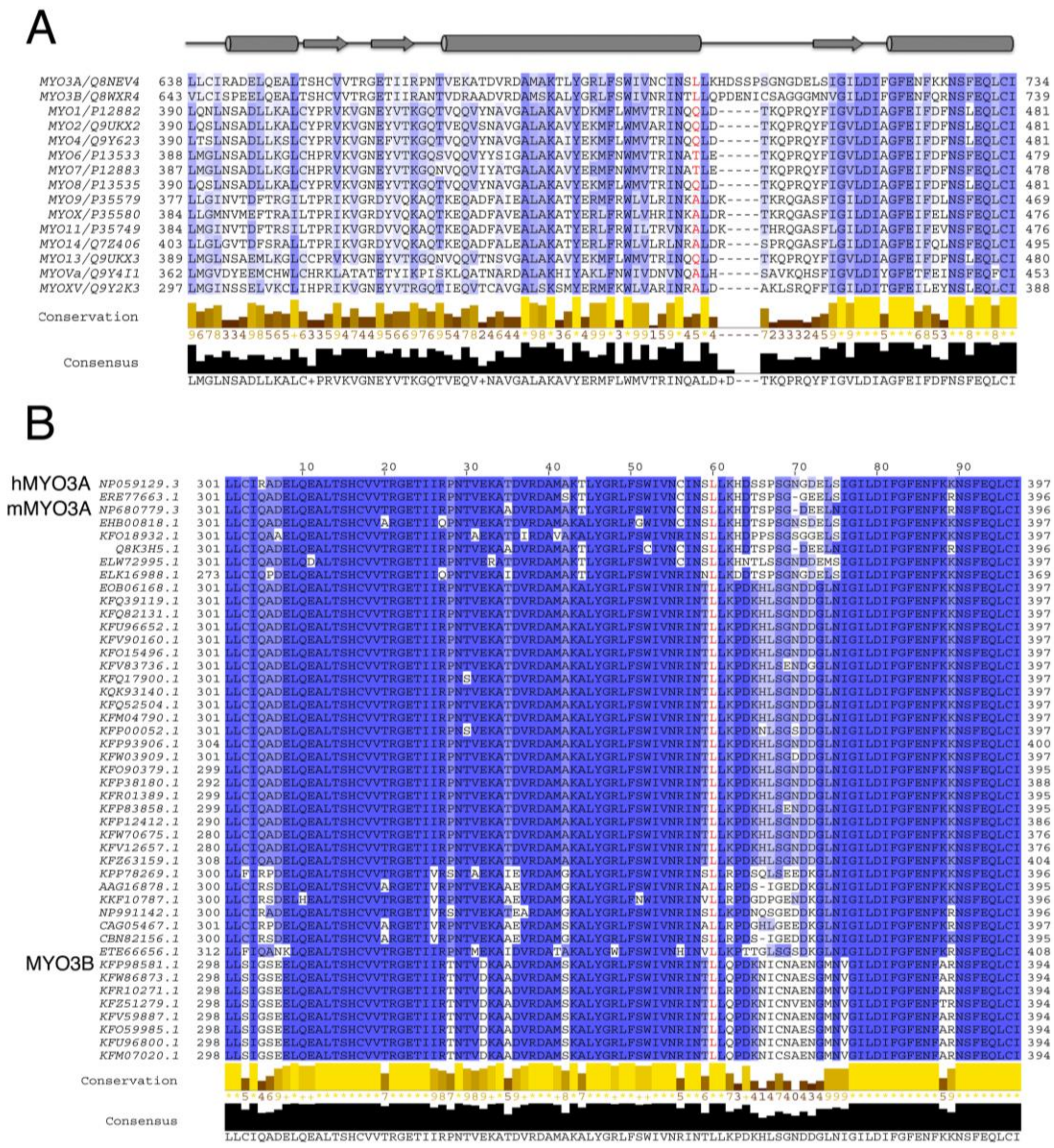

Figure S2: Leucine 697 conservation on MYO3 proteins. (a) Sequence alignment of human myosins colored based on the BLOSUM62 score. Residue 697 is indicated in red. Secondary structure prediction for MYO3A is indicated on top of the alignment with cylinder representing alpha helix, arrows for β-strands and lines for loops. Conservation index for each position are indicated ranging from 9 (highly conserved) to 2 (poorly conserved), fully conserved columns are marked with an asterisk and columns with mutations where all properties are conserved are marked with a '+'. The consensus sequence is shown below the alignment. (b) Sequence alignment of MYO3 proteins colored based on sequence identity showing the conserved residue L697. Human and mouse MYO3A and *Haliaeetus albicilla* MYO3B sequences are indicated. Conservation index for each position is indicated ranging from 9 (highly conserved) to 2 (poorly conserved), fully conserved columns are marked with an asterisk and columns with mutations where all properties are conserved are marked with a '+'. The consensus sequence is shown below the alignment.

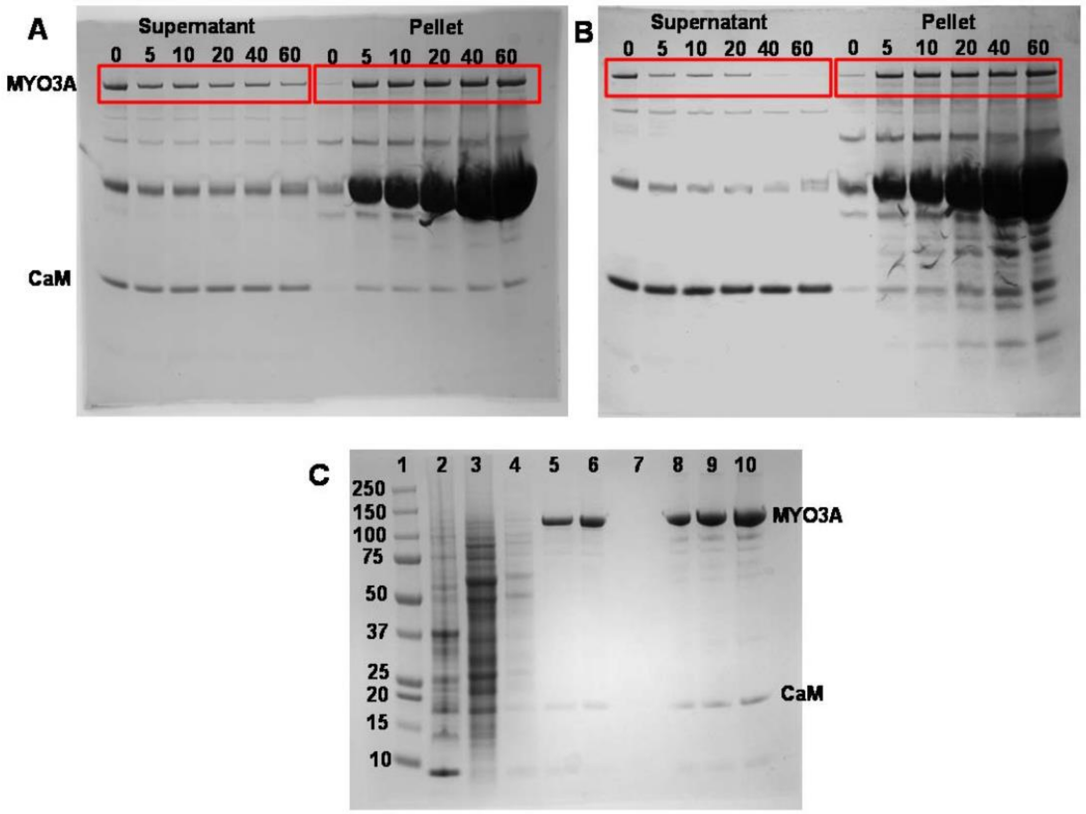

Figure S3: Actin co-sedimentation assay. The SDS gels from the actin co-sedimentation assay are shown for WT (a) and L697W (b) MYO3A. The cropped bands that are included in figure 3e are indicated by the red boxes. c) The molecular weight of MYO3A and calmodulin (CaM) were verified on another SDS gel. Lane 1 is the molecular weight marker and lanes 5, 6, and 8-10 are WT MYO3A containing two IQ domains and a C-terminal GFP (the construct used for this study).

Table S1. Steps in the filtration of exomes from Family 1and from Family 2.

Family 1		List of variants absent in 1000g,ESP, ABraOM and not synonymous		
**Total variants (proband V:4)**	24827	*SELP*	nonsynonymous SNV	NM_003005:c.G170A:p.R57H
**<1% ESP & 1000g**	3811	*MYO3A*	nonsynonymous SNV	NM_017433:c.T2090G:p.L697W
**<1% ABraOM**	1610	*LONP2*	stopgain SNV	NM_031490:c.C1543T:p.Q515X
**Allele Count local cohort (4+62) <= 7**	1077	*HDAC10*	nonsynonymous SNV	NM_001159286:c.T926C:p.L309P
**Family 1: V:34 contains**	96			
**Family 1: V:24 contains**	41			
**Family 1: V:15 contains**	16			
**Absent from 1000g, ESP, ABraOM and not synonymous**	4			
**Previously related to deafness**	1			
**Family 2**		List of variants absent in 1000g,ESP, ABraOM and not synonymous		
**Total variants (proband III:22)**	35417	*MYO3A*	nonsynonymous SNV	MYO3A:NM_017433:exon19:c.T2090G:p.L697W
**<1% ESP & 1000g**	7240	*NACA2*	nonsynonymous SNV	NACA2:NM_199290:exon1:c.C419T:p.A140V
**<1% ABraOM**	3585	*RPTOR*	nonsynonymous SNV	RPTOR:NM_020761:exon16:c.C1712A:p.P571H
**Allele Count local cohort (4+45) <= 7**	1338	*FSCN2*	nonsynonymous SNV	FSCN2:NM_012418:exon1:c.C317T:p.P106L
**Family 2: III:13 contains**	611			
**Family 2: III:3 contains**	269			
**Family 2: IV:8 contains**	75			
**Absent from 1000g, ESP, ABraOM and not synonymous**	4			
**Previously related to deafness**	1			

Table S2. Identical by descendent (IBD) probabilities and relationship between members of the different families. ID represents the identity of the individual in each family, k0 and k1 are the probabilities of sharing 0 or 1 alleles that are IBD, kinship is the kinship coefficient estimated and relationship is the relatedness corresponding to kinship coefficient observed.

ID1	ID2	k0	k1	kinship	Relationship
**Family1 VI:11**	Family2 III:13	0,8806	0,1194	0,0299	
**Family1 VI:11**	Family2 III:19	0,9137	0,0863	0,0216	
**Family1 VI:11**	Family2 IV:11	0,8987	0,1013	0,0253	
**Family1 VI:11**	Family2 V:1	0,9677	0,0323	0,0081	
**Family1 VI:11**	Family2 III:7	0,8845	0,1155	0,0289	
**Family1 VI:16**	Family2 III:13	0,8301	0,1699	0,0425	Second Cousin
**Family1 VI:10**	Family2 III:13	0,7925	0,2075	0,0519	Second Cousin
**Family1 VI:24**	Family2 III:13	0,8638	0,1362	0,0340	Second Cousin
**Family1 VI:19**	Family2 III:13	0,8696	0,1304	0,0326	Second Cousin
**Family1 VI:16**	Family2 III:19	0,8725	0,1275	0,0319	
**Family1 VI:10**	Family2 III:19	0,7693	0,2307	0,0577	Second Cousin
**Family1 VI:24**	Family2 III:19	0,9513	0,0487	0,0122	
**Family1 VI:19**	Family2 III:19	0,9005	0,0995	0,0249	
**Family1 VI:16**	Family2 IV:11	0,8862	0,1138	0,0285	
**Family1 VI:16**	Family2 V:1	0.8420	0.1580	0.0395	Second Cousin
**Family1 VI:16**	Family2 III:7	0.8305	0.1695	0.0424	Second Cousin
**Family1 VI:10**	Family2 IV:11	0.8149	0.1851	0.0463	Second Cousin
**Family1 VI:16**	Family2 V:1	0.8950	0.1050	0.0262	
**Family1 VI:16**	Family2 III:7	0.8314	0.1686	0.0421	Second Cousin
**Family1 VI:24**	Family2 IV:11	0.9873	0.0000	0.0063	
**Family1 VI:24**	Family2 V:1	0.9810	0.0167	0.0053	
**Family1 VI:24**	Family2 III:7	0.9763	0.0237	0.0059	
**Family1 VI:19**	Family2 IV:11	0.9693	0.0248	0.0091	
**Family1 VI:19**	Family2 V:1	0.9638	0.0000	0.0181	
**Family1 VI:19**	Family2 III:7	0.9265	0.0735	0.0184	
